# Spontaneous Splenic Rupture in Atypical Pneumonia From Mycoplasma Infection

**DOI:** 10.7759/cureus.16753

**Published:** 2021-07-30

**Authors:** Karim Nasra, Deep Raole, Neud M Kiros, Alexander Loomis, Eric Rinker

**Affiliations:** 1 Radiology, Ascension Providence Hospital, Southfield, USA; 2 Radiology, Robert Wood Johnson University Hospital, New Brunswick, USA; 3 Radiology, Indiana University School of Medicine, Indianapolis, USA; 4 Interventional Radiology, Ascension Providence Hospital, Southfield, USA

**Keywords:** interventional radiology, embolization, splenic rupture, splenic hemorrhage, minimally invasive

## Abstract

Splenic rupture is a potentially life-threatening condition, often associated with chest or abdominal trauma. Atraumatic splenic rupture (ASR) is quite rare. When reported, it is usually attributed to underlying pathological conditions such as malignant neoplastic disorders, viral infections, or inflammatory processes. Here, we report a case of ASR in a patient that was attributed to *Mycoplasma pneumoniae* infection. This was supported by the diagnosis of atypical pneumonia secondary to *M. pneumoniae*, presence of multiple pseudoaneurysms in the spleen, and presence of cold agglutinins indicating an inflammatory state likely due to systemic vasculitis. Additionally, the lack of recent trauma further corroborated the mycoplasma infection as the driving force behind the splenic rupture.

## Introduction

Splenic rupture is a potentially life-threatening condition, often associated with chest or abdominal trauma. Atraumatic splenic rupture (ASR) is quite rare. When reported, it is usually attributed to underlying pathological conditions such as malignant neoplastic disorders, viral infections, or inflammatory processes.

Here, we report a case of ASR in a patient that was attributed to *Mycoplasma pneumoniae* infection. This was supported by the diagnosis of atypical pneumonia secondary to *M. pneumoniae*, presence of multiple pseudoaneurysms in the spleen, and presence of cold agglutinins indicating an inflammatory state likely due to systemic vasculitis. Additionally, the lack of recent trauma further corroborated the mycoplasma infection as the driving force behind the splenic rupture.

## Case presentation

A 39-year-old male presented to the emergency department for shortness of breath and progressive abdominal pain. He described the abdominal pain as sharp and 8/10 in severity. The pain radiated to the left lower quadrant and worsened with exertion. He denied trauma, hematemesis, or melena. Past medical history included bipolar disorder and remote substance use disorder involving cocaine and opioids. On presentation, heart rate was 130 beats per minute and blood pressure was 126/84. Oxygen saturation was 86% but quickly improved when placed on a 4 L nasal cannula. The patient was afebrile. Physical exam revealed a tender but nonrigid abdomen. Initial labs reported a hemoglobin value of 9.8 gm/dL (previously recorded at 16.0 gm/dL in September 2020) and an elevated white blood cell count at 15.76 K/mcL. Lactate dehydrogenase at 356 unit/L, C reactive protein at 289 mg/L, and D-dimer at 1961 ng/m were all increased. Prothrombin time/international normalized ratio (PT/INR) was within normal limits. The drug screening was negative.

**Figure 1:**
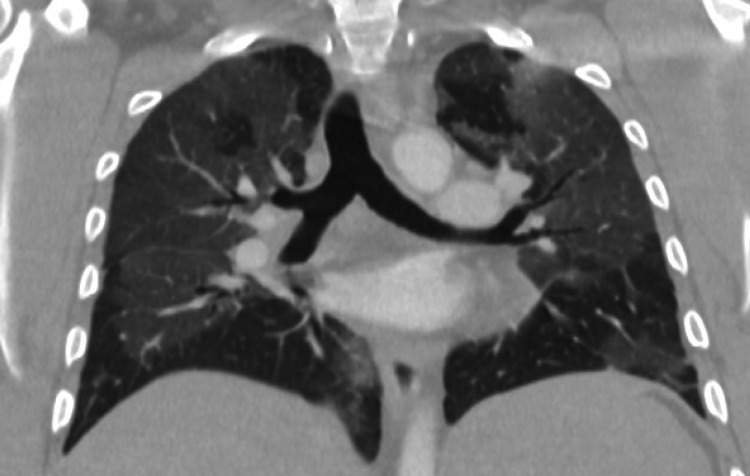
Coronal CT in lung windows shows diffuse ground-glass opacities bilaterally. Findings are overall nonspecific but can be seen with viral/atypical pneumonia.

The initial chest radiograph demonstrated scattered airspace opacities. A CT angiogram (CTA) of the pulmonary arteries was ordered. There was initial concern for pulmonary embolism secondary to hypoxia, tachycardia, and elevated D-dimer. Imaging was extended through the abdomen and pelvis due to abdominal pain and low hemoglobin. The CTA of the pulmonary arteries effectively ruled out pulmonary embolism, however, there were diffuse ground-glass opacities throughout the lungs, which correlated with the plain film radiograph findings (Figure 1). The initial concern was for Covid-19 pneumonia, but the rapid test came back negative. A respiratory virus panel including mycoplasma and Epstein-Barr virus as well as repeat Covid-19 tests was ordered.

**Figure 2:**
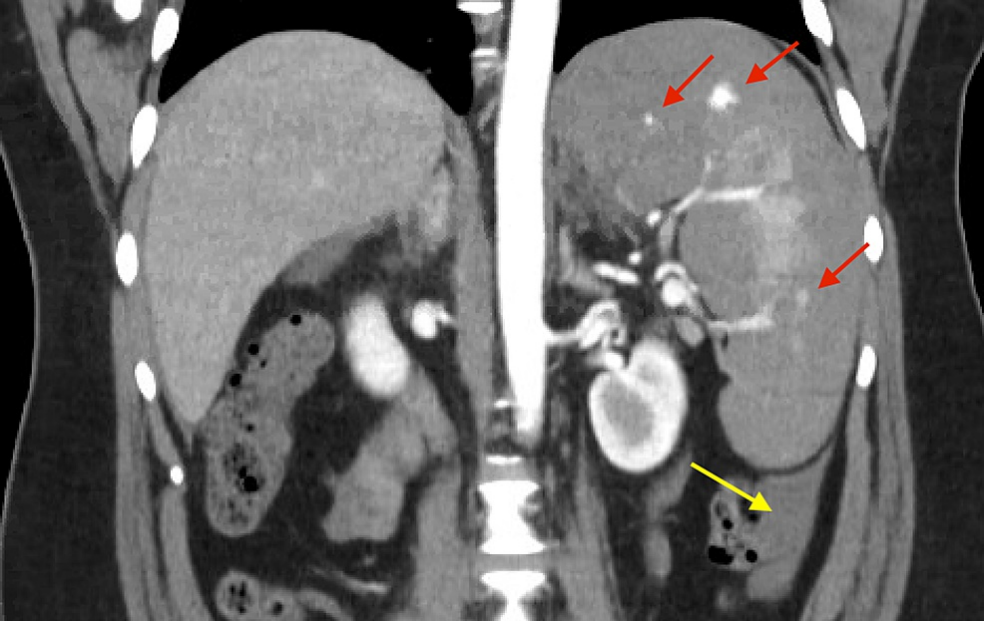
Coronal CT angiography of the abdomen demonstrated multiple areas of active extravasation within the spleen (red arrows). Perisplenic hematoma is observed with hemoperitoneum tracking along the left paracolic gutter (yellow arrow).

CTA of the abdomen demonstrated an enlarged spleen with multiple areas of active extravasation. There was perisplenic hematoma with hyperattenuating fluid tracking down the left paracolic gutter and pooling in the pelvis, compatible with hemoperitoneum (Figure 2). Two units of packed red blood cells and fresh frozen plasma were administered.

**Figure 3:**
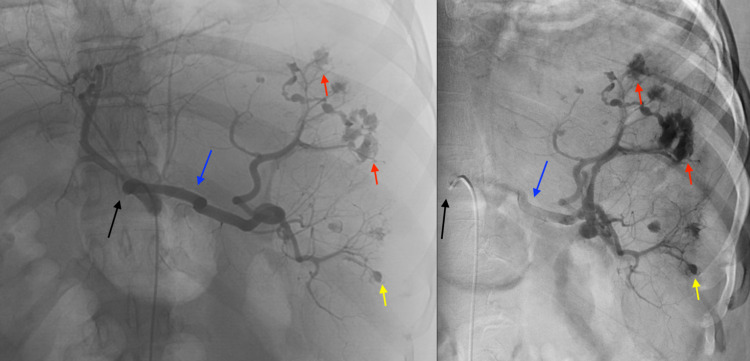
Early (left) and delayed (right) digital subtraction angiography was performed with a catheter in the celiac trunk (black arrow). The splenic artery is well-visualized (blue arrow). There are multiple areas of active hemorrhage, which show increasing extravasation as the run continues (some denoted by the red arrow). There are additional areas of saccular outpouching, which appear unchanged as the run continues compatible with pseudoaneurysm (some denoted by the yellow arrows).

Interventional radiology was consulted for emergent splenic embolization. The patient was screened and taken to the angiographic suite. Antibiotic prophylaxis was administered per protocol with 900 mg of clindamycin and 3 g ancef. Real-time sonographic evaluation of the right groin demonstrated a patent right common femoral artery. Vascular access was gained with a 4 French micropuncture kit and transitioned to a 5 French vascular sheath. Using standard catheter and guidewire technique, the celiac artery was selected with a 5 French Simmons catheter. A celiac arteriogram demonstrated the right hepatic artery arising from the superior mesenteric artery. The common hepatic, left hepatic, gastroduodenal artery, left gastric artery, and splenic arteries were widely patent. The distal splenic arterial branches revealed alternating areas of narrowing and dilatation, simulating a "bead on strings" appearance with multifocal pseudoaneurysms and saccular aneurysms (Figure 3).

A co-axial 3 French microcatheter was used to sequentially subselect the inferior and superior branches of the splenic artery. A digital subtraction arteriogram was performed, which better demonstrated the findings.

Gelfoam (Pfizer, Kalamazoo, Michigan, USA) embolization of the distal splenic arterial branches was then performed. After each administration of Gelfoam slurry, the catheter was pulled back and an arteriogram was performed until there was stagnant flow without visualization of persistent aneurysm or area of extravasation. The catheter was then retracted into the main splenic artery and a subsequent arteriogram reconfirmed satisfactory Gelfoam occlusion.

**Figure 4:**
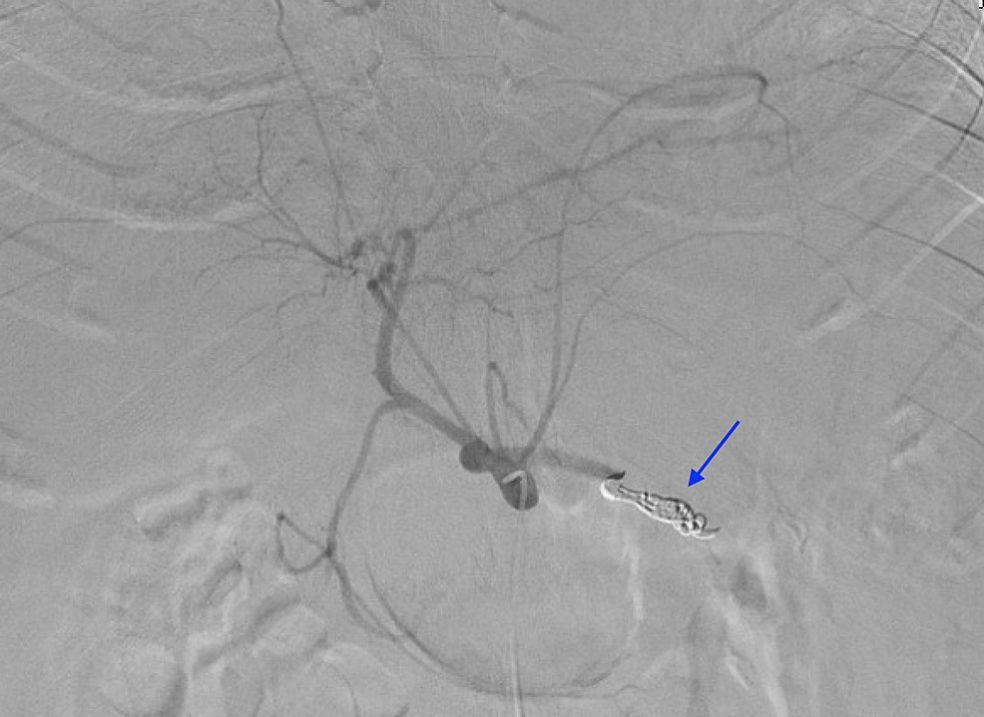
Complete digital subtraction angiogram from the celiac trunk demonstrates satisfactory occlusion of the splenic artery by the coil pack (blue arrow). There is no further evidence of active extravasation or pseduoaneurysm.

The origins of the dorsal pancreatic and pancreatica magna branches from the splenic artery were identified. The catheter was then positioned between these branches and three 8 mm coils were deployed. A celiac arteriogram was performed which confirmed successful coil embolization of the splenic artery with no apparent flow through the coil pack (Figure 4). An arteriogram of the common femoral artery was obtained prior to closure with a Perclose ProGlide device (Abbott Vascular, Redwood City, California, USA). Hemostasis was achieved and dressings were applied. The patient tolerated the procedure well and was transferred to ICU in stable condition.

The patient's hemoglobin stabilized over the next day with overall clinical improvement. During his stay, the respiratory virus panel was positive for mycoplasma titers. After exhausting other etiologies such as trauma, another concurrent infectious process, drug use, or blood dyscrasias, the cause of the patient’s spontaneous splenic hemorrhage was attributed to cold agglutinin disease (CAD) secondary to mycoplasma infection.

The patient was discharged on the post-operative day 3 after receiving a vaccination for *Haemophilus B*, meningococcus, pneumococcus, and influenza.

## Discussion

*Mycoplasma pneumoniae* is a common acute respiratory pathogen that can cause atypical pneumonia, tracheobronchitis, bronchiolitis, and pharyngitis. In addition, a wide spectrum of extrapulmonary manifestations is recognized, including neurological, cardiac, hematologic, renal, and gastrointestinal involvement. The pathogenesis of the extrapulmonary manifestations has been suggested to be an inflammatory basis with transient massive cytokine production and macrophage activation. Opsonization of *M. pneumoniae* particles can elicit an exuberant inflammatory response with varying results depending on the affected organ system [1-2].

A few case reports have described a systemic vasculitis associated with *M. pneumoniae *in the central nervous system as well as in the kidneys [3-5]. The resulting inflammation can weaken the vascular wall leading to aneurysm/pseudoaneurysm formation. This may have been compounded by the patient's remote history of cocaine use, which has a well-documented association with vasculitis. Although rare, isolated atraumatic splenic rupture events have been seen with other vasculitides such as granulomatosis with polyangiitis, polyarteritis nodosa, and systematic lupus erythematosus [6].

Of additional interest to this case is cold agglutinin disease [7]. In cold agglutinin disease, agglutination of red blood cells at below-normal core body temperature causes red blood cell breakdown via extravascular hemolysis [8]. The spleen identifies these agglutinated red blood cells as abnormal and breaks them down at an increased rate which can overwhelm the spleen, induce splenomegaly, and increase the risk for splenic rupture.

When a patient presents with atraumatic splenic rupture, the decision for management depends on multiple factors including hemodynamic stability and the extent of splenic injury. It is necessary to consider whether the cause is due to a systemic process or limited to the spleen. Options range from total splenectomy, spleen-preserving surgery, trans-arterial embolization, to conservative management. In our case, relative patient stability and CTA findings of active bleeding and hemoperitoneum led to urgent angiography and embolization with the intention of preserving as much splenic parenchyma as possible. Although the underlying etiology was not discovered until later, it would not have changed management. Even if the patient had been symptomatically anemic from CAD, splenectomy is unlikely to be effective as the liver is the main site of hemolysis in CAD. One of the benefits of a total splenectomy is the ability for histological tissue analysis to establish a diagnosis. Further, if the spleen is already hypo- or dysfunctional due to the same inciting pathology that leads to rupture, a splenectomy would not necessarily increase the risk of post-splenectomy infection [9].

## Conclusions

Atraumatic splenic rupture is an uncommon condition that is often confounded by the fact that a definite inciting cause is not always found. The most common etiologies are hematological disorders, infections (such as this case), or localized inflammatory processes such as pancreatitis or colitis. If a cause such as a malignant infiltrative process can be deciphered beforehand, the patient should proceed straight to total splenectomy. In the emergent setting without an immediately available etiology, a conservative approach with maximal preservation of functional splenic tissue is favored.
